# Individual differences in looking at persons in scenes

**DOI:** 10.1167/jov.22.12.9

**Published:** 2022-11-07

**Authors:** Maximilian Davide Broda, Benjamin de Haas

**Affiliations:** 1 Experimental Psychology, Justus Liebig University, Giessen, Germany; 2 Center for Mind, Brain and Behavior (CMBB), University of Marburg and Justus Liebig University, Giessen, Germany

**Keywords:** individual differences, person saliency, gaze behavior, scene perception

## Abstract

Individuals freely viewing complex scenes vary in their fixation behavior. The most prominent and reliable dimension of such individual differences is the tendency to fixate faces. However, much less is known about how observers distribute fixations across other body parts of persons in scenes and how individuals may vary in this regard. Here, we aimed to close this gap. We expanded a popular annotated stimulus set (Xu, Jiang, Wang, Kankanhalli, & Zhao, 2014) with 6,365 hand-delineated pixel masks for the body parts of 1,136 persons embedded in 700 complex scenes, which we publish with this article (https://osf.io/ynujz/). This resource allowed us to analyze the person-directed fixations of 103 participants freely viewing these scenes. We found large and reliable individual differences in the distribution of fixations across person features. Individual fixation tendencies formed two anticorrelated clusters, one for the eyes, head, and the inner face and one for body features (torsi, arms, legs, and hands). Interestingly, the tendency to fixate mouths was independent of the face cluster. Finally, our results show that observers who tend to avoid person fixations in general, particularly do so for the face region. These findings underscore the role of individual differences in fixation behavior and reveal underlying dimensions. They are further in line with a recently proposed push–pull relationship between cortical tuning for faces and bodies. They may also aid the comparison of special populations to general variation.

## Introduction

Humans constantly move their eyes to counteract crowding and limited acuity outside the fovea and process detailed information from different image regions (Gegenfurtner, 2016; Rosenholtz, 2016). Saliency models aim to predict which features of a given scene will attract gaze. Several models, like the classic one by Itti, Koch, and Niebur (1998) rely on low-level features such as the local contrast for color, intensity or orientation. However, later models have shown that fixation behavior typically is dominated by high-level factors such as the presence of objects (Einhäuser, Spain, & Perona, 2008; Kümmerer, Theis, & Bethge, 2015) and their size (Borji, Sihite, & Itti, 2013), the spatial distribution of meaning (Henderson & Hayes, 2017) and semantic content (Xu et al., 2014) in a scene.

Certain semantic categories, such as faces outweigh others in their saliency (Cerf, Paxon Frady, & Koch, 2009; Xu et al., 2014). Faces and especially eyes elicit faster saccades than other stimuli (Broda, Haddad, & de Haas, 2022; Crouzet, Kirchner, & Thorpe, 2010) and most observers fixate faces within the first two fixations when present in a scene. Consequently, the addition of face detection to low-level saliency models significantly improves gaze prediction (Cerf, Harel, J., Einhäuser, & Koch, 2008). On top of the general tendency to fixate faces, there are large and reliable individual differences (Guy et al., 2019). This individual tendency to fixate faces correlates with face recognition abilities and a range of other semantic salience biases (de Haas, Iakovidis, Schwarzkopf, & Gegenfurtner, 2019; Linka, Broda, Alsheimer, Haas, & Ramon, 2022; Linka & de Haas, 2020). At least for autism spectrum disorders, the tendency to (not) fixate faces may also be linked to a general (lack of) social attention for people (Riby & Hancock, 2008). Furthermore, individuals show distinct preferences in the way they fixate faces. Special populations such as observers with congenital prosopagnosia (Avidan & Behrmann, 2021) or autism spectrum disorders (Reimann et al., 2021; Tanaka & Sung, 2016) may show distinct fixation profiles within faces. Stable individual differences have also been shown for healthy controls, specifically regarding the preferred vertical landing position of face directed saccades. Although most observers prefer fixating close to the eye region, the preferred landing position of some can be as far down as the mouth. These preferences appear individually optimal (Peterson & Eckstein, 2013) and highly stable, generalizing from screen-based lab experiments to real world interactions (Peterson, Lin, Zaun, & Kanwisher, 2016). However, it is unclear whether and how these individual biases in face fixations relate to other facets of gaze behavior. As mentioned above, in autism spectrum disorder the tendencies to fixate eyes, faces and people may be linked to each other (Riby & Hancock, 2008; Tanaka & Sung, 2016). Furthermore, individuals with exceptional abilities in face recognition and a strong tendency to fixate faces, also tend to fixate consistently closer to the theoretically optimal position just below the eyes (Linka, Broda, et al., 2022; Peterson & Eckstein, 2012). Whether and how the individual way of fixating faces and people is linked to social salience in the general population has yet to be determined.

Although faces are extremely salient, they rarely occur isolated within a scene and most often as part of a human body. However, much less is known about the saliency of other body parts during free viewing. Just as faces, depictions of whole persons can elicit rapid saccades (Fletcher-Watson, Findlay, Leekam, & Benson, 2008). Social features and emotional content have been shown to influence gaze behavior when freely viewing complex images or dynamic scenes (End & Gamer, 2017; Rubo & Gamer, 2018). To evaluate social information, bodies play an important role, especially in the interpretation of emotional expressions (Aviezer, Trope, & Todorov, 2012). Consequently, gaze behavior is adapted toward the emotional content of a scene. Negative social interactions elicit more body and less face fixations compared with positive social interactions (McFarland et al., 2013). Body fixations may also be subject to individual differences. For instance, which bodies and body parts a given observer fixates for how long may be modulated by their sex (Lykins, Meana, & Strauss, 2008; Nummenmaa, Hietanen, J. K., Santtila, & Hyönä, 2012). Additionally, recent research has found large and reliable differences in the tendency to fixate objects being touched, which were anticorrelated with individual face salience and may point to individual differences in hand saliency (de Haas et al., 2019).

Recent evidence from macaques reinforce the idea of a push–pull mechanism between the preference for faces and other body parts. Specifically, face-deprived monkeys show little interest in faces while paying more attention to hands compared with controls (Arcaro, Schade, P. F., Vincent, Ponce, & Livingstone, 2017). Differences in visual experience also modulate semantic saliency biases in humans. Preschool children fixate faces and hands significantly more often than adults but are less likely to fixate text elements in complex natural scenes (Linka, Sensoy, Karimpur, Schwarzer, & de Haas, 2022). However, the saliency of different body parts for persons in scenes as well as potential related individual differences have yet to be studied systematically.

Here, we introduce a total of 6,365 hand-delineated pixel masks allowing the precise mapping of nine different body parts (arms, hands, torsi, legs, heads, inner faces, mouths, eyes and background bodies) for 1,136 persons embedded in 700 complex scenes used in previous eye tracking studies (Xu et al., 2014). We make this resource publicly available (https://osf.io/ynujz/) and use it to explore human gaze behavior toward persons in scenes. Specifically, we determine the relative salience of bodily and facial features in complex scenes and investigate the extent and covariance pattern of related individual differences. Briefly, our findings show that eyes and mouths by far attract the most fixations relative to pixel size. At the same time, observers strongly and reliably vary in their tendency to fixate different person features. These fixation tendencies systematically covary and predominantly fall into two anticorrelated clusters—head, inner face and eyes on the one hand and body features on the other. The tendency to fixate mouths is largely independent from either cluster. Finally, our results show that observers who (in comparison with others) tend to avoid person fixations particularly do so for the face region.

## Methods

### Subjects

We re-analyzed a previously published dataset (Linka & de Haas, 2020). The dataset consists of fixation data from 103 participants (age: *M* = 25 years; *SD* = 6; 72 females) who participated in two sessions with an average delay of 16 days between, acquired at the Leibniz Institute for Psychology Trier. All participants had normal or corrected to normal vision. The study was approved by the local ethics committee at Justus Liebig University Giessen, and conducted in accordance with the declaration of Helsinki. All participants gave informed consent before the experiment. For details, see Linka and de Haas (2020).

### Stimuli and pixel masks

**Figure 1.**
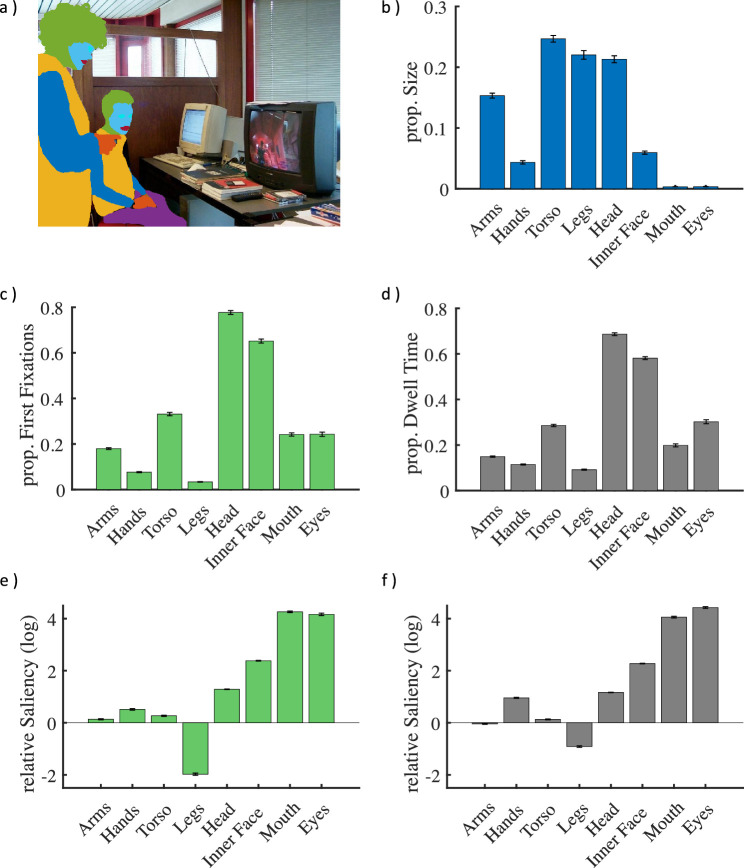
Fixation attraction and pixel size of different features. (a) Depiction of a stimulus with overlayed pixel masks for arms (dark blue), hands (dark orange), torsi (light orange), legs (purple), heads (green), inner faces (light blue), mouths (red) and eyes (cyan), and (b) their pixel size relative to the respective person, averaged across all instances in the stimulus set. Bar plots in (c–f) depict (c) the proportions of first fixations and (d) proportional dwell times. Note that the sum of fixation proportions is larger than 1 because of (1) overlapping features such as head, inner face, mouth, and eyes and (2) the possibility of a fixation falling on multiple features (the diameter was defined as 1° visual angle). Panels (e) and (f) show the log scaled ratio of fixation proportions over relative pixel sizes for (e) first fixations and (f) dwell times. Note that zero corresponds to the expected value for randomly distributed fixations. Error bars show standard error of the mean across observers (SEM).

We used the Object and Semantic Images and Eye-tracking (*OSIE*) set. The stimulus set is publicly available and consists of 700 natural scenes (https://www-users.cse.umn.edu/∼qzhao/predicting.html) with hand-drawn pixel masks and categorical semantic labels for 5,551 objects. For details, see Xu et al. (2014). Crucially, we created 6,365 additional hand-delineated pixel masks and labels (Figure 1a) for arms (841), hands (741), torsi (875), legs (647), heads (1018), inner faces (735), mouths (649), eyes (671), and background bodies whose feature parts were not distinguishable (188). We also created separate person pixel masks that combined all features belonging to a particular person.

### Age and gender ratings

Five naïve participants who did not complete the experiment (age: *M* = 22 years; *SD* = 2; 4 females) rated each's depicted person's age and gender, though only persons with existing pixel masks for inner faces and eyes and/or mouth were considered. In total, participants rated 680 persons. We included the mean age estimate for each person after excluding values that were above or below one standard deviation of the combined ratings. Gender was included if no contradicting judgments were made. All divergent judgments could be resolved after additional inspection (52 persons). These ratings are publicly available with the remaining pixel masks but are not further analyzed in this article.

### Apparatus

Participants placed their head in a chin and forehead rest and viewed stimuli at a distance of 64 cm at 29.7° × 22.3° visual angle (dva). The experiment was controlled via Psychtoolbox (Kleiner et al., 2007) and Matlab (MathWorks, Natick, MA). Gaze data were acquired from the participant's right eye using an EyeLink 1000 Plus eye tracker (SR Research, Ottawa, Canada) at a frequency of 2 kHz.

### Procedure

During the first session, participants freely viewed all 700 images, which were presented in the same order for all. Images were split into 7 blocks of 100 each. Participants could pause the experiment and leave the chin and forehead rest in between blocks as a nine-point (re)calibration was done before the start of each block (validation error: *M* = 0.35° [dva], *SD* = 0.12 dva). Each trial started with a central fixation and participants could trigger the 3-second image presentation via button press. The complete experiment lasted between 60 and 90 minutes. After an average of 16 days, participants completed the procedure again with a subset of 200 images (two blocks; validation error: *M* = 0.35 dva, *SD* = 0.17 dva).

### Analysis

For the present study, we analyzed fixations which fell on the following features: arms, hands, torsi, legs, heads, inner faces, mouths, and eyes. We excluded fixations on background bodies (bodies without distinguishable features) from our analysis because of their small number and our interest in distinguishable features.

#### Cleaning and labeling

Fixations were labeled if they landed on a labeled person feature or were within a distance of 0.5 dva from the respective feature mask. Note that a radius of 0.5 dva is within foveal resolution. Consequently, a given fixation can fall on multiple features in the scene. All fixations earlier than 100 ms after image onset (onset fixations) and shorter than 100 ms in duration were excluded from further analyses. First fixations were defined as the first fixation in each trial (excluding onset fixations).

#### Relative feature size

The relative feature size was calculated as the number of pixels for a given feature mask, divided by the sum of pixels for the respective person mask (i.e., all features). If a given feature was not visible (for instance, if the legs of a person were not shown), the relative size was coded as zero. Relative feature sizes were then averaged across all persons.

#### Proportion of first fixations

The proportion of first fixations was defined by adding the number of cases in which the first fixation after image onset landed on a given feature, divided by the number of cases the first fixation landed on any person feature. This procedure was done for each observer and person feature, separately for all, odd and even trials. Additionally, we calculated the proportion of first fixations landing on any person feature across all trials for each observer.

#### Proportion of dwell time

The proportion of dwell time was defined as the proportion of dwell time (across all fixations that landed on person features) that a given participant spent on a given feature. Again, this was calculated separately for each feature in all, odd and even trials. Additionally, we calculated the proportion of overall dwell time (across all fixations) a given individual spent on person features.

#### Relative saliency

We calculated the relative saliency for each feature by dividing individual fixation proportions for a given feature (across all person fixations in all images) by its average relative pixel size. This procedure was done for all proportions of first fixations and dwell times. Note that (ignoring the fixation tolerance radius) this metric has an expected value of 1 for randomly distributed fixations and 0 after log scaling.

#### Split-half consistencies

We assessed the reliability of individual differences in fixation behavior by correlating individual fixation proportions across odd and even trials, separately for each feature. To quantify the range of individual differences, we computed the ratio of the maximum over the minimum individual proportion for each feature.

#### Test–retest reliability 

Test–retest reliability was assessed by correlating individual fixation proportions across all trials between session 1 and session 2. Note that the resulting estimates are lower boundaries of retest reliability because participants completed 469 trials containing person features during the first session, but only a subset of 155 person trials during the second session (Linka & de Haas, 2020). Therefore, only images that were shown in both sessions were included in this analysis. All other analyses were limited to and included all data from the first session.

#### Covariance patterns

Individual fixation tendencies toward different semantic features can covary systematically (de Haas et al., 2019). To test this for person features, we computed zero-order correlations between individual fixation proportions for all features, separately for first fixations and dwell times. We then subjected the resulting correlation matrices to multidimensional scaling to project them onto a two-dimensional plane and visualize their structure. To rule out the possibility of a calibration artefact accounting for our results, we limited our analyses to the features of only the 30%, 20%, and 10% largest person masks in the scenes and correlated the resulting covariance patterns with the original. Because faces usually appear in the upper image halve and bodies in the lower, possible fixation preferences for these features (especially for the first saccade after image onset) could reflect idiosyncratic visual field biases. To test whether semantic salience biases persist independent of such visual field effects, we quantified the amount of face and body pixels falling in the upper and lower image halves and tested the consistency of individual fixation biases toward heads and bodies across upper and lower image halves. Specifically, we correlated the proportions of first fixations falling onto heads in the upper image halve with that of first fixations falling onto heads in the lower image halve and similarly the proportions of first fixations onto bodies across upper and lower image halves.

#### Social salience correlations

Social salience correlations were defined as the correlation between the tendency to look at a given feature within persons (i.e., the proportion of feature fixations among person fixations) and the tendency to look at persons in general (i.e., the proportion of all fixations directed toward persons). Again, this was computed separately for first fixations and dwell times.

All correlations were calculated using Pearson's correlation coefficient. Significance was determined at a family-wise error rate (FWE) of α = 0.05 using the Holm–Bonferroni method to correct for multiple testing (reported *p* values are uncorrected, but only marked as significant if they survived FWE correction).

## Results

### Relative saliency

To determine the relative saliency of features we divided the proportion of person-directed fixations a given feature attracted by the average proportion of person pixels the feature comprises. We opted for this straightforward measure because it is readily interpretable—the expected value for random fixation distributions (ignoring the tolerance) is 1—and directly relates to the proportions of fixations we used for individual differences analyses (as discussed elsewhere in this article). Please note that the sum of proportional fixations is greater than 1 because of overlapping features in the head region (e.g., eyes within the inner face). Additionally, the applied tolerance of 0.5 dva enabled the possibility of a single fixation falling on multiple features.

First, we calculated the pixel size of a given feature relative to that of the whole person and computed the average of this relative size for each category. Torsi (*M* = 0.25) and legs (*M* = 0.22) had the highest relative pixel size, followed by heads (*M* = 0.21), arms (*M* = 0.15), inner faces (*M* = 0.06), hands (*M* = 0.04), eyes (*M* < 0.01), and mouths (*M* < 0.01). Note that the sum of relative sizes is less than 1 owing due to the fact that not all features are visible for every depicted person (Figure 1b). Even though body features tend to have a greater relative pixel size, participants preferably looked at the face features head (first, *M* = 0.78; dwell, *M* = 0.69), inner face (first, *M* = 0.66; dwell, *M* = 0.58) and eyes (first, *M* = 0.24; dwell, *M* = 0.30) followed by torso (first, *M* = 0.33; dwell, *M* = 0.29), mouth (first, *M* = 0.24; dwell, *M* = 0.20), arms (first, *M* = 0.18; dwell, *M* = 0.15), hands (first, *M* = 0.08; dwell, *M* = 0.11), and legs (first, *M* = 0.03; dwell, *M* = 0.09) (Figures 1c–d). Accordingly, the inner face features eyes (first, *M* = 70.59; dwell, *M* = 87.87) and mouth (first, *M* = 74.29; dwell, *M* = 61.08) possessed the greatest relative salience, whereas the relative salience was smaller for inner faces (first, *M* = 10.95; dwell, *M* = 9.77), heads (first, *M* = 3.64; dwell, *M* = 3.22), and hands (first, *M* = 1.77; dwell, *M* = 2.65) and close to or below the chance level for torsi (first, *M* = 1.34; dwell, *M* = 1.16), arms (first, *M* = 1.17; dwell, *M* = 0.97), and legs (first, *M* = 0.15; dwell, *M* = 0.42) (Figures 1e–f).

### Individual differences: Split-half consistencies and covariance patterns

**Figure 2.**
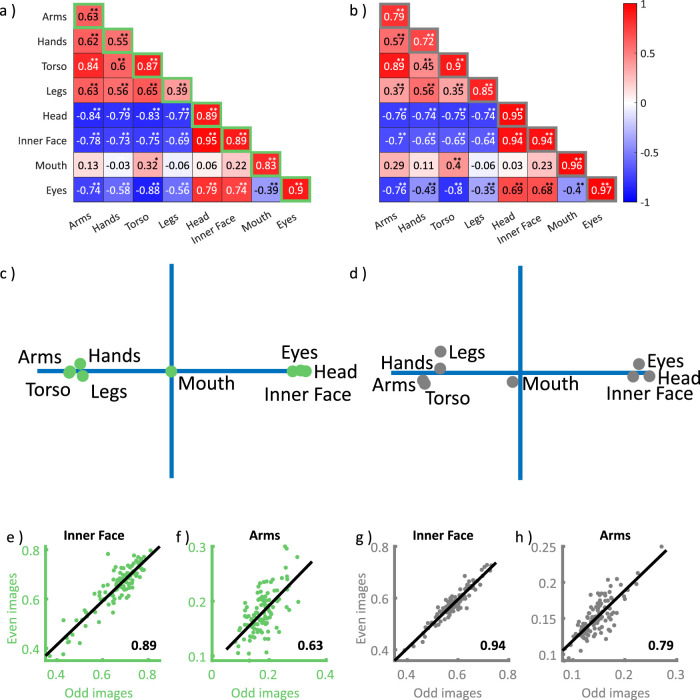
Split-half consistencies and interfeature correlations. Correlation matrices in panels (a–b) show the covariance between fixation tendencies in off-diagonal cells and their split-half consistencies on the diagonal for (a) proportions of first fixations and (b) proportional dwell times. Negative to positive correlations are indicated by color and saturation, as shown on the color bar to the right. Asterisks indicate statistical significance (Holm-Bonferroni corrected for multiple testing) **p < 0.001, *p < 0.05. Panels (c–d) show the corresponding two-dimensional projection derived with multidimensional scaling for the proportions of (c) first fixations and (d) dwell times. Panels (e–h) show example scatter plots for consistency correlations (diagonal cells in a–b). Each dot represents the proportion of first fixations (e–f) or dwell times (g–h) of a single observer, which landed on the respective feature in odd versus even trials. Least square lines are shown in grey and the corresponding correlation values in the bottom right of each plot.

Participants showed large variability in their tendency to fixate different person features. The greatest differences were observed for the tendency to direct the first fixation directed at a person onto the eyes—with a 10-fold range across observers—and for the proportion of person-dwell time falling onto mouths—ranging 6-fold across observers (Table 1). These differences proved consistent for all features when analyzing split-half correlations between odd and even trials for the proportions of first fixations (Figures 2a, e–f) and dwell times (Figures 2b, g–h). Split-half correlations ranged from *r* = 0.39 (*p* < 0.001) (legs) to *r* = 0.90 (*p* < 0.001) (eyes) for proportions of first fixations and between *r* = 0.72 (*p* < 0.001) (hands) and *r* = 0.97 (*p* < 0.001) (eyes) for proportional dwell times (diagonal values in Figures 2a–b).

**Table 1.** Maximum over minimum ratios for proportions of first fixations and proportional dwell times.

	Arms	Hands	Torso	Legs	Head	Inner face	Mouth	Eyes
First fixations	2.96	5.47	2.75	7.53	1.81	2.18	6.02	9.81
Dwell time	2.98	2.39	2.77	3.72	1.53	1.86	6.07	4.96

Correlating fixation tendencies across features revealed two clusters of positive intercorrelations, the first between bodily features (arms, hands, torso, and legs) and the second between all facial features but the mouth (head, inner face, eyes) for both, proportions of first fixations (all *r* > 0.5) and dwell times (all *r* > 0.3). The tendency to fixate the mouth only showed a moderate correlation with the tendency to fixate torsi (first, *r* = 0.32, *p* = 0.001, uncorrected; dwell, *r* = 0.40, *p* < 0.001) and a negative correlation with the tendency to fixate eyes (first, *r* = −0.39, *p* < 0.001; dwell, *r* = −0.40, *p* < 0.001). The tendencies to fixate bodily and facial features in turn were anticorrelated with each other (first, all *r* < −0.5; dwell, all *r* < −0.3) (Figures 2a–b; see Supplements S1 for bootstrapped split-half correlations). This pattern was reflected in a two-dimensional projection based on multidimensional scaling, which indicated two distinct clusters for the tendency to fixate bodily (arms, hands, torso, and legs) and facial (head, inner face, and eyes) features as polar opposites along the first dimension, with the tendency to fixate mouths falling in between (Figures 2c–d). To further rule out the possibility of a calibration artefact accounting for our results, we limited our analyses to the features of only the 30%, 20%, and 10% largest person masks in the scenes. The covariance patterns yielded by the original and size restricted analyses were highly consistent and all correlated with r ≥ 0.9, ruling out calibration artefacts as a source of the observed pattern.

Further control analyses revealed that less than 12% of all face pixels are in the lower visual field. Similarly, only 25% of all body pixels appear in the upper visual field. This implies a low number of possible first fixations onto these features at atypical visual field locations. Despite this lacking power, first fixation tendencies for faces were significantly correlated across the upper and lower image halves (*r* = 0.26, *p* = 0.007), as were those for bodies (*r* = 0.33, *p* < 0.001).

### Individual differences: Test–retest reliability

**Figure 3.**
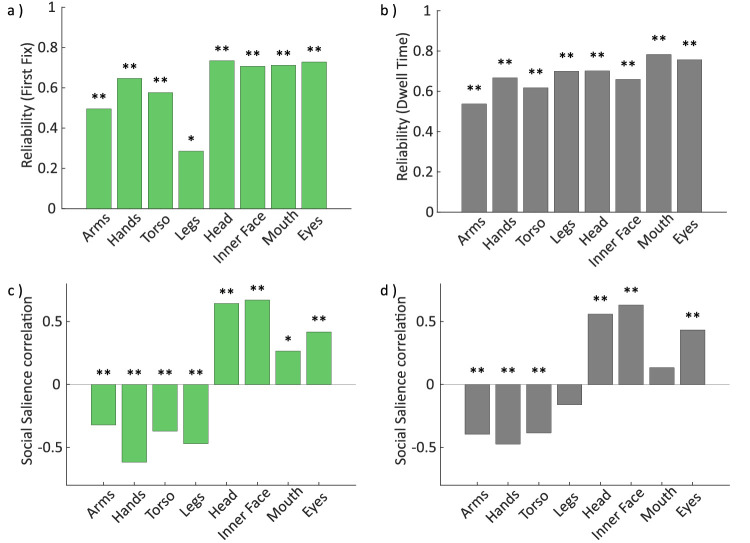
Test–retest reliability and social salience correlations. (a–b) Test–retest reliability of individual differences in the proportions of (a) first fixations and (b) dwell times landing on a given feature. All tendencies showed moderate to high reliability across the two sessions. (c–d) Social salience correlations between the individual proportion of fixations landing on persons overall and the distribution of these person-directed fixations across features for (c) first fixations and (d) dwell time. Results indicate that participants who looked more often at persons overall also tended to direct more of these fixations onto the face and fewer onto the body. Asterisks indicate statistical significance (Holm-Bonferroni corrected for multiple testing) **p < 0.001, *p < 0.05.

The test–retest reliability of individual fixation tendencies was moderate to high for all features (all *r* ≥ 0.5) (Figures 3a–b), indicating their stability across sessions with the exception of proportions of first fixation toward legs (*r* = 0.29, *p* = 0.004). This was true for both, first fixations (ranging from *r* = 0.50, *p* < 0.001 for arms to *r* = 0.73, *p* < 0.001 for heads) (Figure 3a) and dwell times (ranging from *r* = 0.54, *p* < 0.001 for arms to *r* = 0.78, *p* < 0.001 for mouths) (Figure 3b).

### Individual differences: Person saliency

Finally, we tested whether individual differences in the overall tendency to look at persons correlated with differences in the distribution of these fixations across features. Indeed, the tendency to fixate persons correlated positively with the tendency to direct person fixations toward facial features (head, inner face, and eyes) and negatively with the tendency to direct person fixations to bodily features (arms, hands, torso, and legs). Specifically, the tendency to direct the fixation within a scene onto a person was significantly anticorrelated with the tendency to direct the first fixation onto a given person toward their arms (*r* = −0.32, *p* < 0.001), hands (*r* = −0.61, *p* < 0.001), torso (*r* = −0.37, *p* < 0.001) or legs (*r* = −0.47, *p* < 0.001), but positively correlated with the tendency to direct this fixation to the head (*r* = 0.64, *p* < 0.001), inner face (*r* = 0.67, *p* < 0.001), eyes (*r* = 0.42, *p* < 0.001), and mouth (*r* = 0.27, *p* = 0.007) (Figure 3c). Similarly, the proportion of overall dwell time spent on persons was significantly anticorrelated with the tendency to allocate person directed dwell time on arms (*r* = −0.39, *p* < 0.001), hands (*r* = −0.47, *p* < 0.001) and torsi (*r* = −0.39, *p* < 0.001), but positively correlated with the tendency to dwell on heads (*r* = 0.56, *p* < 0.001), inner faces (*r* = 0.63, *p* < 0.001), and eyes (*r* = 0.43, *p* < 0.001) (no significant correlations with legs, *r* = −0.16, *p* = 0.107, or mouth, *r* = 0.13, *p* = 0.174) (Figure 3d).

## Discussion

The goal of this study was to quantify and compare the salience of different person features in complex scenes and to test potential related individual differences. We created hand-delineated pixel masks for arms, hands, torsi, legs, heads, inner faces, mouths, and eyes of 1,136 persons embedded in 700 complex scenes (Xu et al., 2014) that were freely viewed by 103 participants (Linka & de Haas, 2020).

Our analyses revealed that, even though body features such as torsi and legs had the largest share of person pixels in the images, face regions attracted more fixations, underscoring their saliency (Cerf et al., 2008, 2009; de Haas et al., 2019). Relative to pixel size, eyes and mouths attracted almost 10 times as many fixations as any other person feature, underscoring the salience of inner face features (Barton, Radcliffe, Cherkasova, Edelman, & Intriligator, 2006; Birmingham, Bischof, & Kingstone, 2008; Kauffmann, Khazaz, Peyrin, & Guyader, 2021; van Belle, Ramon, Lefèvre, & Rossion, 2010; Vo, Smith, Mital, & Henderson, 2012). After the head and face region, human torsi attracted most fixations relative to pixel size, possibly reflecting their importance for judgments regarding attractiveness (Dixson, Grimshaw, Linklater, & Dixson, 2011, 2014) and emotional state (Aviezer et al., 2012).

Beyond the group level, observers strongly and reliably varied in their tendency to fixate person features and particularly in their tendency to fixate facial features (approximately 2-fold for faces and approximately 10-fold for eyes across 103 observers). This outcome is in line with previous findings showing that face saliency varies across observers (de Haas et al., 2019; Guy et al., 2019) and that observers vary in their preferred vertical fixation location within faces (Linka, Broda, et al., 2022; Peterson et al., 2016; Peterson & Eckstein, 2013). Importantly, here we extend these findings to other body parts. We found individual differences in fixation tendencies for all person features, which were consistent across images and reliable across time, indicating their trait like nature (de Haas et al., 2019).

Additionally, we found strong intercorrelations between fixation tendencies for most face and body features, which fell into three distinct clusters—anticorrelated tendencies to either fixate the body or the head (including the inner face and eyes) and a largely independent tendency to fixate the mouth. Interestingly, the tendency to fixate facial features correlated with general social salience—that is, the overall proportion of fixations landing on persons. At the same time, the tendency to fixate bodies (rather than the head) was anticorrelated with social salience. This finding suggests that observers who (in comparison to others) tend to avoid person fixations in scenes, particularly do so for the face region. Given observers shift fixations from the face region to the body when scene images depict negative (compared with positive) social interactions (McFarland et al., 2013), future studies should test whether observers with low person and face salience generally assign lower relevance to social stimuli.

Covariance patterns between fixation tendencies have previously been shown for other sematic features. Participants with a strong tendency to fixate faces are less likely to look at text within static scenes and vice versa. Similar results were obtained for the tendency to fixate faces and objects being touched, which was interpreted as a potential push-pull mechanism between the individual salience for faces and hands (de Haas et al., 2019). Our present results confirm reliable individual differences in hand salience, which are indeed strongly anticorrelated with individual face salience. This echoes the finding that macaques growing up face deprived show decreased salience for faces and an increased tendency to fixate hands. These face-deprived macaques also showed decreased neuronal preferences for faces and enlarged cortical hand patches (Arcaro et al., 2017). Similarly, recent findings suggest that face and word-selective patches of human ventral cortex developmentally expand at the expense of limb-preferring areas (Nordt et al., 2021), echoing salience differences between preschool children and adults (Linka, Sensoy, et al., 2022). Our present findings add to this converging evidence for a push–pull relationship between face and limb salience and show that it generalizes to (almost) all body parts as well as to individual differences between adults. Future studies should test whether individual differences in human gaze behavior are reflected in the functional layout of the ventral stream in the individual brain.

An open question in this context is to which degree individual differences in viewing behavior toward static scenes generalize to real-world vision. Previous research found that humans situationally adapt their viewing behavior toward moving faces. Specifically, observers are more likely to look at the eye region when the depicted person looks at the camera while talking increases the number of mouth fixations (Vo et al., 2012). At the same time, a recent study found that individual biases in person viewing generalize from static to dynamic scene viewing (Broda & de Haas, 2022) and individual fixation behavior toward static faces is highly predictive of gaze behavior during real world interactions (Peterson et al., 2016). Additionally, even screen-based gaze behavior that does not generalize to real-world interactions may have diagnostic value in clinical contexts (Adolph & West, 2022). Nevertheless, future research should test to which degree the individual biases we document here generalize to real-world interactions.

A related, interesting question is to which degree individual semantic salience is tied to spatial oculomotor or visual field biases. Faces typically appear in the upper halves of a scene and the remaining body parts in the lower halves. Previous studies have found idiosyncratic visual field biases (Afraz, Pashkam, & Cavanagh, 2010; Fortenbaugh, Silver, & Robertson, 2015; Greenwood, Szinte, Sayim, & Cavanagh, 2017; Kwon & Liu, 2019; Moutsiana et al., 2016), which could contribute to the individual biases we observed. However, despite only a small proportion of faces and bodies falling into the lower and upper image halves, respectively, an additional control analysis revealed significant consistency of individual face and body salience across the upper and lower image halves. Future research should investigate the role of visual field and oculomotor biases on free viewing behavior using dedicated experiments and stimulus material optimized to tease them apart.

Our finding that general social salience correlates with the tendency to fixate the inner face region and especially the eye region adds to research on gaze behavior and social cognition. Previous studies reported that autism spectrum disorder is associated with avoidance of social stimuli and the eye region of human faces (Bird, Press, & Richardson, 2011; Chita-Tegmark, 2016; Klin, Jones, Schultz, Volkmar, & Cohen, 2002; Tanaka & Sung, 2016; but see Reimann et al., 2021 for a counterexample). Some studies also describe an increased tendency to fixate the mouth (Klin et al., 2002; Neumann, Spezio, Piven, & Adolphs, 2006) but this remains controversial (Guillon, Hadjikhani, Baduel, & Rogé, 2014). Observers with autism spectrum disorder have shown problems assessing emotional cues from faces which might result from the avoidance of eye regions. Our data suggest that neurotypical participants who avoid faces and eyes show a generally reduced tendency to fixate persons. Previous results have shown that the individual tendency to fixate faces corelates with face recognition abilities (de Haas et al., 2019; Linka, Broda, et al., 2022). It would be interesting to test further potential functional consequences of individual differences in social and face salience, for example, in evaluating social stimuli or emotional expressions. In general, inner face features have shown to be a very important source of information in the context of face identification (Peterson & Eckstein, 2012; Schyns, Bonnar, & Gosselin, 2002) and facial expressions (Smith, Cottrell, Gosselin, & Schyns, 2005).

In conclusion, we find that inner face features are by far the most salient features of persons in scenes. At the same time, observers show large (up to 10-fold) and reliable individual differences the way they distribute person-directed fixations across features. These individual differences covary in a structured manner, falling into three distinct clusters: anticorrelated tendencies to either fixate the body or the head (including the eyes and inner face) and a separate tendency to fixate the mouth. Observers with an increased tendency to fixate the body rather than the face, show a generally reduced tendency to fixate persons. This finding has profound theoretical implications, because it reveals a low-dimensional space of gaze preferences toward body parts, narrows the search space for underlying mechanisms, echoes the cortical clustering and potential competition of preferences found in the extrastriate body and face patches (Arcaro et al., 2017), and provides new evidence for the relationship between general social and face salience. We hope the publicly available study material that led to these findings will prove a helpful resource for further studies into the salience of people in scenes.

## Supplementary Material

Supplement 1

## References

[bib1] Adolph, K. E., & West, K. L. (2022). Autism: The face value of eye contact. *Current Biology,* 32(12), R577–R580, 10.1016/J.CUB.2022.05.016. 35728531 PMC9527854

[bib2] Afraz, A., Pashkam, M. V., & Cavanagh, P. (2010). Spatial heterogeneity in the perception of face and form attributes. *Current Biology,* 20(23), 2112–2116, 10.1016/J.CUB.2010.11.017. 21109440 PMC5056559

[bib3] Arcaro, M. J., Schade, P. F., Vincent, J. L., Ponce, C. R., & Livingstone, M. S. (2017). Seeing faces is necessary for face-domain formation. *Nature Neuroscience,* 20(10), 1404–1412, 10.1038/nn.4635. 28869581 PMC5679243

[bib4] Avidan, G., & Behrmann, M. (2021). Spatial integration in normal face processing and its breakdown in congenital prosopagnosia. Annual Review of Vision Science*,* 7, 301–321, 10.1146/annurev-vision-113020-012740. 34014762

[bib5] Aviezer, H., Trope, Y., & Todorov, A. (2012). Body cues, not facial expressions, discriminate between intense positive and negative emotions. *Science,* 338(6111), 1225–1229, 10.1126/science.1224313. 23197536

[bib6] Barton, J. J. S., Radcliffe, N., Cherkasova, M. v., Edelman, J., & Intriligator, J. M. (2006). Information processing during face recognition: The effects of familiarity, inversion, and morphing on scanning fixations. *Perception,* 35(8), 1089–1105, 10.1068/p5547. 17076068

[bib7] Bird, G., Press, C., & Richardson, D. C. (2011). The role of alexithymia in reduced eye-fixation in autism spectrum conditions. *Journal of Autism and Developmental Disorders,* 41(11), 1556–1564, 10.1007/s10803-011-1183-3. 21298331

[bib8] Birmingham, E., Bischof, W., & Kingstone, A. (2008). Gaze selection in complex social scenes. *Visual Cognition,* 16(2–3), 341–355, 10.1080/13506280701434532.

[bib9] Borji, A., Sihite, D. N., & Itti, L. (2013). What stands out in a scene? A study of human explicit saliency judgment. *Vision Research,* 91, 62–77, 10.1016/j.visres.2013.07.016. 23954536

[bib10] Broda, M. D., & de Haas, B. (2022). Individual fixation tendencies in person viewing generalize from images to videos. *i-Perception,* 12(2), 1–10, 10.1177/20416695211009552. PMC9638695 36353505

[bib11] Broda, M. D., Haddad, T., & de Haas, B. (2022). *Quick, eyes! Isolated upper face halves but not artificial features elicit rapid saccades*. PsyArXiv, 5 July, 10.31234/OSF.IO/24GSJ. PMC9919614 36749582

[bib12] Cerf, M., Harel, J., Einhäuser, W., & Koch, C. (2008). Predicting human gaze using low-level saliency combined with face detection. *Advances in Neural Information Processing Systems,* 20, 241–248.

[bib13] Cerf, M., Paxon Frady, E., & Koch, C. (2009). Faces and text attract gaze independent of the task: Experimental data and computer model. *Journal of Vision,* 9(12), 1–15, 10.1167/9.12.10. 20053101

[bib14] Chita-Tegmark, M. (2016). Social attention in ASD: A review and meta-analysis of eye-tracking studies. Research in Developmental Disabilities*,* 48, 79–93, 10.1016/j.ridd.2015.10.011. 26547134

[bib15] Crouzet, S. M., Kirchner, H., & Thorpe, S. J. (2010). Fast saccades toward faces: Face detection in just 100 ms. *Journal of Vision,* 10(4), 1–17, 10.1167/10.4.16. 20465335

[bib16] de Haas, B., Iakovidis, A. L., Schwarzkopf, D. S., & Gegenfurtner, K. R. (2019). Individual differences in visual salience vary along semantic dimensions. *Proceedings of the National Academy of Sciences of the United States of America,* 116(24), 11687–11692, 10.1073/PNAS.1820553116. 31138705 PMC6576124

[bib17] Dixson, B. J., Grimshaw, G. M., Linklater, W. L., & Dixson, A. F. (2011). Eye-tracking of men's preferences for waist-to-hip ratio and breast size of women. *Archives of Sexual Behavior,* 40(1), 43–50, 10.1007/s10508-009-9523-5. 19688590

[bib18] Dixson, B. J., Grimshaw, G. M., Ormsby, D. K., & Dixson, A. F. (2014). Eye-tracking women's preferences for men's somatotypes. *Evolution and Human Behavior,* 35(2), 73–79, 10.1016/j.evolhumbehav.2013.10.003.

[bib19] Einhäuser, W., Spain, M., & Perona, P. (2008). Objects predict fixations better than early saliency. *Journal of Vision,* 8(14), 1–26, 10.1167/8.14.18. 19146319

[bib20] End, A., & Gamer, M. (2017). Preferential processing of social features and their interplay with physical saliency in complex naturalistic scenes. *Frontiers in Psychology,* 8, 418, 10.3389/fpsyg.2017.00418. 28424635 PMC5371661

[bib21] Fletcher-Watson, S., Findlay, J. M., Leekam, S. R., & Benson, V. (2008). Rapid detection of person information in a naturalistic scene. *Perception,* 37(4), 571–583, 10.1068/p5705. 18546664

[bib22] Fortenbaugh, F. C., Silver, M. A., & Robertson, L. C. (2015). Individual differences in visual field shape modulate the effects of attention on the lower visual field advantage in crowding. *Journal of Vision,* 15(2), 19–19, 10.1167/15.2.19. PMC4327314 25761337

[bib23] Gegenfurtner, K. R. (2016). The interaction between vision and eye movements. Perception*,* 45, (12), 1333–1357, 10.1177/0301006616657097. 27383394

[bib24] Greenwood, J. A., Szinte, M., Sayim, B., & Cavanagh, P. (2017). Variations in crowding, saccadic precision, and spatial localization reveal the shared topology of spatial vision. *Proceedings of the National Academy of Sciences of the United States of America,* 114(17), E3573–E3582, 10.1073/PNAS.1615504114. 28396415 PMC5410794

[bib25] Guillon, Q., Hadjikhani, N., Baduel, S., & Rogé, B. (2014). Visual social attention in autism spectrum disorder: Insights from eye tracking studies. Neuroscience and Biobehavioral Reviews*,* 42, 279–297, 10.1016/j.neubiorev.2014.03.013. 24694721

[bib26] Guy, N., Azulay, H., Kardosh, R., Weiss, Y., Hassin, R. R., Israel, S., & Pertzov, Y. (2019). A novel perceptual trait: Gaze predilection for faces during visual exploration. *Scientific Reports,* 9(1), 1–12, 10.1038/s41598-019-47110-x. 31341217 PMC6656722

[bib27] Henderson, J. M., & Hayes, T. R. (2017). Meaning-based guidance of attention in scenes as revealed by meaning maps. *Nature Human Behaviour,* 1(10), 743–747, 10.1038/s41562-017-0208-0. PMC7455012 31024101

[bib28] Itti, L., Koch, C., & Niebur, E. (1998). A model of saliency-based visual attention for rapid scene analysis. *IEEE Transactions on Pattern Analysis and Machine Intelligence,* 20(11), 1254–1259, 10.1109/34.730558.

[bib29] Kauffmann, L., Khazaz, S., Peyrin, C., & Guyader, N. (2021). Isolated face features are sufficient to elicit ultra-rapid and involuntary orienting responses toward faces. *Journal of Vision,* 21(2), 1–24, 10.1167/jov.21.2.4. PMC7873494 33544121

[bib30] Kleiner, M., Brainard, D., Pelli, D., Ingling, A., Murray, R., & Broussard, C. (2007). What's new in Psychtoolbox-3? *Perception,* 36, 1–16. ECVP Abstract Supplement.

[bib31] Klin, A., Jones, W., Schultz, R., Volkmar, F., & Cohen, D. (2002). Visual fixation patterns during viewing of naturalistic social situations as predictors of social competence in individuals with autism. *Archives of General Psychiatry,* 59(9), 809–816, 10.1001/archpsyc.59.9.809. 12215080

[bib32] Kümmerer, M., Theis, L., & Bethge, M. (2015). Deep gaze I: Boosting saliency prediction with feature maps trained on ImageNet. *Conference on Learning Representations, ICLR*. May 7–9. San Diego, California.

[bib33] Kwon, M. Y., & Liu, R. (2019). Linkage between retinal ganglion cell density and the nonuniform spatial integration across the visual field. *Proceedings of the National Academy of Sciences of the United States of America,* 116(9), 3827–3836, 10.1073/PNAS.1817076116. 30737290 PMC6397585

[bib34] Linka, M., Broda, M. D., Alsheimer, T., de Haas, B., & Ramon, M. (2022). Characteristic fixation biases in Super-Recognizers. *Journal of Vision,* 22(8), 1–17, 10.1167/JOV.22.8.17. PMC9344214 35900724

[bib35] Linka, M., & de Haas, B. (2020). OSIEshort: A small stimulus set can reliably estimate individual differences in semantic salience. *Journal of Vision,* 20(9), 1–9, 10.1167/JOV.20.9.13. PMC7509791 32945849

[bib36] Linka, M., Sensoy, Ö., Karimpur, H., Schwarzer, G., & de Haas, B. (2022). *Attentional biases in free viewing of complex scenes in preschoolers and adults*. PsyArXiv. April 19, 10.31234/osf.io/x59vr. PMC10362043 37479760

[bib37] Lykins, A. D., Meana, M., & Strauss, G. P. (2008). Sex differences in visual attention to erotic and non-erotic stimuli. *Archives of Sexual Behavior,* 37(2), 219–228, 10.1007/s10508-007-9208-x. 17668312

[bib38] McFarland, R., Roebuck, H., Yan, Y., Majolo, B., Li, W., & Guo, K. (2013). Social interactions through the eyes of macaques and humans. *PLoS One,* 8(2), e56437, 10.1371/journal.pone.0056437. 23457569 PMC3574082

[bib39] Moutsiana, C., de Haas, B., Papageorgiou, A., van Dijk, J. A., Balraj, A., Greenwood, J. A., & Schwarzkopf, D. S. (2016). Cortical idiosyncrasies predict the perception of object size. *Nature Communications,* 7(1), 1–12, 10.1038/ncomms12110. PMC4931347 27357864

[bib40] Neumann, D., Spezio, M. L., Piven, J., & Adolphs, R. (2006). Looking you in the mouth: Abnormal gaze in autism resulting from impaired top-down modulation of visual attention. *Social Cognitive and Affective Neuroscience,* 1(3), 194–202, 10.1093/scan/nsl030. 18985106 PMC2555425

[bib41] Nordt, M., Gomez, J., Natu, V. S., Rezai, A. A., Finzi, D., Kular, H., & Grill-Spector, K. (2021). Cortical recycling in high-level visual cortex during childhood development. *Nature Human Behaviour,* 5(12), 1686–1697, 10.1038/s41562-021-01141-5. PMC8678383 34140657

[bib42] Nummenmaa, L., Hietanen, J. K., Santtila, P., & Hyönä, J. (2012). Gender and visibility of sexual cues influence eye movements while viewing faces and bodies. *Archives of Sexual Behavior,* 41(6), 1439–1451, 10.1007/s10508-012-9911-0. 22402995

[bib43] Peterson, M. F., & Eckstein, M. P. (2012). Looking just below the eyes is optimal across face recognition tasks. *Proceedings of the National Academy of Sciences of the United States of America,* 109(48), E3314–E3323, 10.1073/pnas.1214269109. 23150543 PMC3511732

[bib44] Peterson, M. F., & Eckstein, M. P. (2013). Individual differences in eye movements during face identification reflect observer-specific optimal points of fixation. *Psychological Science,* 24(7), 1216, 10.1177/0956797612471684. 23740552 PMC6590077

[bib45] Peterson, M. F., Lin, J., Zaun, I., & Kanwisher, N. (2016). Individual differences in face-looking behavior generalize from the lab to the world. *Journal of Vision,* 16(7), 12, 10.1167/16.7.12. 27191940

[bib46] Reimann, G. E., Walsh, C., Csumitta, K. D., McClure, P., Pereira, F., Martin, A., & Ramot, M. (2021). Gauging facial feature viewing preference as a stable individual trait in autism spectrum disorder. *Autism Research,* 14(8), 1670–1683, 10.1002/aur.2540. 34008916 PMC10277907

[bib47] Riby, D. M., & Hancock, P. J. B. (2008). Viewing it differently: Social scene perception in Williams syndrome and autism. *Neuropsychologia,* 46(11), 2855–2860, 10.1016/j.neuropsychologia.2008.05.003. 18561959

[bib48] Rosenholtz, R. (2016). Capabilities and limitations of peripheral vision. Annual Review of Vision Science*,* 2, 437–457, 10.1146/annurev-vision-082114-035733. 28532349

[bib49] Rubo, M., & Gamer, M. (2018). Social content and emotional valence modulate gaze fixations in dynamic scenes. *Scientific Reports,* 8(1), 1–11, 10.1038/s41598-018-22127-w. 29491440 PMC5830578

[bib50] Schyns, P. G., Bonnar, L., & Gosselin, F. (2002). Show me the features! Understanding recognition from the use of visual information. *Psychological Science,* 13(5), 402–409, 10.1111/1467-9280.00472. 12219805

[bib51] Smith, M. L., Cottrell, G. W., Gosselin, F., & Schyns, P. G. (2005). Transmitting and decoding facial expressions. *Psychological Science,* 16(3), 184–189, 10.1111/j.0956-7976.2005.00801.x. 15733197

[bib52] Tanaka, J. W., & Sung, A. (2016). The “eye avoidance” hypothesis of autism face processing. *Journal of Autism and Developmental Disorders,* 46(5), 1538–1552, 10.1007/s10803-013-1976-7. 24150885 PMC3997654

[bib53] van Belle, G., Ramon, M., Lefèvre, P., & Rossion, B. (2010). Fixation patterns during recognition of personally familiar and unfamiliar faces. *Frontiers in Psychology,* 1, 20, 10.3389/fpsyg.2010.00020. 21607074 PMC3095380

[bib54] Vo, M. L. H., Smith, T. J., Mital, P. K., & Henderson, J. M. (2012). Do the eyes really have it? Dynamic allocation of attention when viewing moving faces. *Journal of Vision,* 12(13), 1–14, 10.1167/12.13.3. 23211270

[bib55] Xu, J., Jiang, M., Wang, S., Kankanhalli, M. S., & Zhao, Q. (2014). Predicting human gaze beyond pixels. *Journal of Vision,* 14(1), 1–20, 10.1167/14.1.28. 24474825

