# Supplementary material for: Individual differences in looking at persons in scenes
Source: J Vis. 2022 Nov 7;22(12):9. doi: 10.1167/jov.22.12.9 (PMC9652713; doi:10.1167/jov.22.12.9)
Supplement: Supplement 1 [file jovi-22-12-9_s001.pdf]

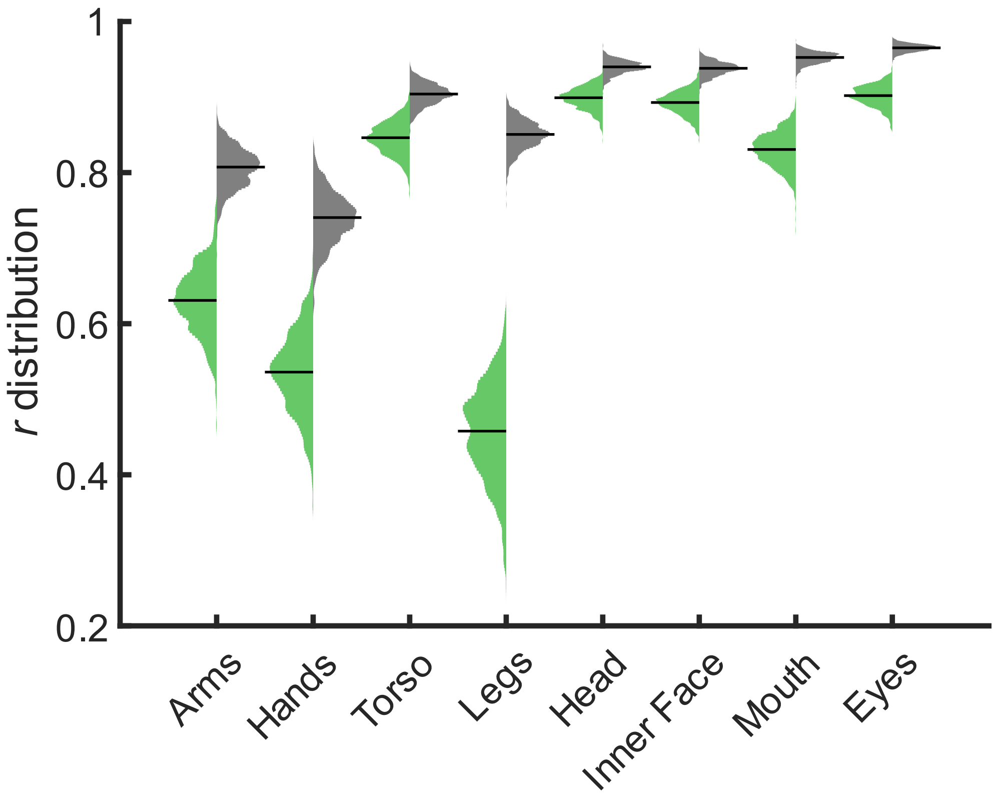

Figure S1 Distribution of bootstrapped split-half correlations

Histograms of split-half correlations for 1000 random splits for all features colored in green and grey for proportions of first fixations and proportional dwell times, respectively. Black lines indicate the median consistency correlation for each distribution. Median values range from r = .46 (Legs) to r = .90 (Eyes) for proportions of first fixations (Arms: r = .63; Hands: r = .54; Torso: r = .85; Head: r = .90; Inner Face: r = .89; Mouth: r = .83) and from r = .74 (Hands) to r = .97 (Eyes) for proportional dwell times (Arms: r = .81; Torso: r = .90; Legs: = .85; Head: r = .94; Inner Face: r = .94; Mouth: r = .95).
